# Knowledge and Attitudes about Mother-to-Child Transmission of the Human Immunodeficiency Virus in a Context of Social Vulnerability: The Case of the Province of Essaouira, Morocco

**DOI:** 10.4314/ejhs.v33i3.3

**Published:** 2023-05

**Authors:** Nezha Nacer, Nadia Ouzennou, Najat El Fatimi, Samia Rkha

**Affiliations:** 1 Laboratory of Pharmacology, Neurobiology, Anthropobiology, Environment and Behavior, Biology, Faculty of Sciences Semlalia, Cadi Ayyad A University, Marrakech, Morocco; 2 Higher Institute of Nursing and Health Techniques of Marrakech, Marrakech, Morocco; 3 Doctor, Health Province of Essaouira, Essaouira, Morocco

**Keywords:** Health knowledge, attitudes, and practices, Preventive measures, Mother-to-child transmission, HIV, HIV infections, Pregnant women

## Abstract

**Background:**

Despite health education efforts, pregnant women still face major health problems.The aim of this study was to assess the knowledge and attitudes of pregnant women on measures to prevent mother-to-child transmission of the human immunodeficiency virus in a context of social vulnerability.

**Methods:**

This is a cross-sectional survey of 384 pregnant women randomly selected from the 20 health districts in the province of Essaouira. This study was carried out from January 2022 to June 2022. A questionnaire was used, and bivariable logistic regression analyses were done to identify factors associated with knowledge and attitudes of pregnant women about mother-to-child transmission of the human immunodeficiency virus.

**Results:**

Low knowledge (75.8%) and negative attitudes (72.7%) about preventing mother-to-child transmission of HIV were observed in study participants. The knowledge and attitudes of pregnant women about the prevention of mother-to-child transmission of HIV varied by education level, number of children, and source of information. There was a very significant correlation between knowledge and attitude (p=0.000).

**Conclusion:**

A significant number of pregnant women have incomplete knowledge and attitudes about preventing mother-to-child transmission of HIV. Actions to increase the knowledge of pregnant women are essential. The capacity of healthcare providers should also be strengthened to improve the quality of care.

## Introduction

Preventing mother-to-child transmission (PMTCT) eliminating new pediatric human immunodeficiency virus (HIV) infection (1). Human immunodeficiency virus (HIV) in children result of vertical transmission, also known as mother-to-child transmission (PMT). Globally, not one of the 2018 or 2020 targets for increased acceleration to eradicate the AIDS epidemic among children and adolescents has been met, a situation compounded by the Covid-19 pandemic (2). In Morocco, in 2020, a total of 850 children are on triple antiretroviral therapy (ART), which represents 5% of people living with HIV on treatment (3). HIV infection in children shows an infection in the parents, and its identification is usually done at an advanced stage of the disease, sometimes making the prognosis unfavorable (4). In this sense, preventing mother-to-child transmission (PMTCT) of HIV from an HIV-positive mother to her child during pregnancy, labor, childbirth, or breastfeeding is a public health issue (5). In the absence of any intervention at these stages, transmission rates can range from 15% to 45% (6, 7). By administering antiretrovirals to mothers and infants as early as possible in pregnancy and breastfeeding, mother-to-child transmission can be almost completely avoided (7). Without treatment, half of the children living with HIV die before the age of two (8), with a peak in mortality between 2 and 3 months for newborns (9). This vertical transmission is very high in resource-limited countries, making prevention of mother-to-child transmission (PMTCT) a priority intervention for HIV/AIDS programs and remaining a challenge in the majority of these countries, especially in Africa (10). Indeed, since the initiation of PMTCT, one million deaths and 2.2 million HIV infections have been prevented among children, achieving the goal. The current focus is the elimination of all new HIV infections in children and ensuring the survival of their mothers (2). In this context, Morocco has committed to formally integrating HIV counseling and testing for pregnant women and their spouses or partners into the minimum package of prenatal consultation services (public and private sectors). Its extension in primary health care (PHC) facilities will provide 95% of pregnant women access to HIV testing and, where appropriate, antiretroviral therapy (11). In this sense, the success of such a prevention strategy depends on informing the target subjects, in this case, pregnant women (12). The challenges to be anticipated in this context are the knowledge and attitudes of expectant mothers towards the available preventive measures for HIV/AIDS PMTCT. This knowledge collaborates with the attitude—the way or feeling of someone like a pregnant woman on HIV/AIDS PMTCT services—to achieve better results. It is therefore worth noting that the level of knowledge and attitudes towards HIV/AIDS PMTCT prevention measures and their adequacy may not be the same around the world. Indeed, ten European countries and the United States of America reported 85% knowledge of HIV/AIDS prevention (12). Simultaneously, results of a study revealed that the proportion of women with comprehensive knowledge of HIV/AIDS MTCT prevention in Nigeria was 7.5% of the national population, while that of Mali and Lesotho was 10.3% and 11.8%, respectively (13). The percentage of women who could identify all HIV/AIDS preventive measures according to the five modes of HIV/AIDS transmission was high in European countries of Belarus (34.98%) and Ukraine (31.67%) which reveals their positive attitude towards the services (14). Attitudes determine the adoption of PMTCT services. Several studies conducted in different parts of the world found that the uptake of PMTCT services was 53% in North America, 62% in Europe, 63% in South America, 84% in Africa, and 84% in Asia (15). In Morocco, pregnant women aspire to have healthy, HIV/AIDS-free newborns. Nevertheless, it is assumed that Moroccan pregnant women do not have good knowledge and attitudes towards HIV/AIDS MTCT prevention measures to achieve their wishes. This is due to many barriers, including low screening coverage in antenatal clinics (ANCs), delivery houses, and hospital maternity wards and low awareness of the value of HIV/AIDS MTCT prevention measures (16). Moreover, a survey conducted among health professionals on knowledge, attitudes, and practices related to HIV/AIDS in children in the University Hospital Center (CHU) of Marrakech (17) revealed a lack of knowledge of MTCT and PMTCT of HIV/AIDS. Only 38.05% of providers knew the initiation period of antiretroviral treatment in children, and the proposal of HIV testing in pregnant women is not systematic according to 53.98% of respondents. It is thus assumed that expectant mothers in the province of Essaouira in Morocco are not exempt from this situation. Based on these findings and in the absence of studies on the subject, this study aimed to investigate the knowledge and attitudes about preventive measures to protect pregnant women from mother-to-child transmission of HIV/AIDS in the province of Essaouira in Morocco.

Thus, the most important research questions of this study were articulated as follows:

1. To what extent are pregnant women informed about the prevention of mother-to-child transmission of HIV?
2. What kind of attitudes do pregnant women have toward PMTCT interventions and services?

## Materials and Methods

**Setting of the study**: The study was carried out in the province of Essaouira, which is part of the Marrakech-Safi region; a city on the Atlantic Ocean located 174 km west of the city of Marrakech and 173 km north of Agadir. The province had a total population of 77,966 inhabitants in 2014, divided into two axes, the Haha and Chiadma. It is characterized by a rural predominance of 77.6%. It was the fifth poorest province in Morocco, recording a poverty rate of 22.1% (8.2% in Morocco) (18). The study was conducted from January 2022 to June 2022 in the 20 health districts of the province.

**Design**: The present study is a descriptive cross-sectional study of pregnant women in the Moroccan region of Essaouira. The sample size was determined on the basis of estimates of antenatal visit targets in the 20 health facilities where the trial was conducted, representing 54382 pregnant women. Thus, 490 pregnant women constituted the required sample size for this study with a 95% confidence level, and a margin of error of 5%.

In this study, stratified random sampling was used to identify the number of participants based on the number of pregnant women served by each health center. Subsequently, based on a list provided by the health authorities, participants were selected by simple random sampling until the target number per center was reached. Thus, 490 pregnant women were involved in the study. Subsequently, the pregnant women concerned in each health district were called on the day of data collection. The participation rate was 78.4%. Therefore, 384 pregnant women were interviewed.

Prior to the administration of the questionnaire, participants were provided with all the necessary information about the study before signing an informed consent form. A structured questionnaire developed by the authors was used after the care or services for which the pregnant women had come to the center. It targeted the sociodemographic, socioeconomic and health-characteristics of the women concerned. In addition, it provided information on knowledge and attitudes about prevention of mother-to-child transmission of HIV. This knowledge and attitude was based on the WHO (2009) PMTCT program components, including HIV counseling and testing, antiretroviral treatment and prophylaxis, safer delivery practices, safer feeding practices, and referral of HIV-positive mothers and infants to treatment, care and support services. For face validity, the tool was first tested, refined, and given to experts in the field.

In this study, sufficient knowledge about PMTCT was defined as knowledge of: (1) the three times of HIV transmission from mother to child (during pregnancy, delivery, and breastfeeding); (2) how to prevent vertical transmission of HIV, and (3) when to administer antiretrovirals in newborns (within 72 hours). Attitudes toward PMTCT were defined by the following elements: acceptance of early HIV testing in every pregnancy and spousal testing; adherence to PMTCT program activities (management if positive, spousal care, protection, and care of the newborn). The questionnaire was constructed using a five-point Likert scale. The target population approves each of the proposals with one of the following ratings: very unfavorable, unfavorable, indifferent, favorable and very favorable. Each possible approval has its own rating.

**Data analysis:** The data collected was checked and analyzed using the Statistical Package for Social Studies for Windows (SPSS version 18). The accuracy of the data was carefully checked before analysis. Statistical processing included calculation of frequencies, means, and standard deviations. Chi-square test to capture associations between categorical variables. Binary logistic regression to remove confounders and capture the weight of variables associated with knowledge and attitudes toward mother-to-child transmission of HIV. Statistical significance was set at the 5% level.

**Ethical approval**: An authorization from the ethics committee [01/REC/22] of the Moroccan Association for Research and Ethics, Polydisciplinary Faculty of Taroudant as well as an authorization for data collection from the health authorities: the regional health directorate of the Marrakech-Safi region, were obtained. The participants gave their voluntary and informed consent to participate in this study. This means that participation in the research respects the autonomy of the women, who have the right to participate or not, and to freely withdraw at any time. In addition, they were previously informed about the nature of the study, the authors, the objectives, and the confidentiality of the data collected.

## Results

**Sociodemographic and health profiles of the women studied:** Data were obtained from interviews with the 384 pregnant women, supplemented by their medical records. The sociodemographic and health profiles are presented in Table 1. Most of the participants in this study were young (mean age: 30.65 years, SD = 8.1), with extremes ranging from 18 to 49 years. The illiteracy rate was 49.2% and 89.3% were from a monogamous marriage. One hundred and seventy-two (44.8%) had no social security coverage and 50.8% lived in rural areas. The majority were in the inactive category (86.7%). In Morocco, annual family income varies from 0 to 32,000 dirhams, with an average of 38,167 dirhams (Moroccan currency, or 4,246.85 US dollars (USD) (18).

**Table 1:** Distribution of pregnant women (n=384) by socio-demographic and health characteristics

Variables	Modalities	Number (%)
**Age groups**	15-24 yrs old	113 (29.4)
	25-34 yrs old	144 (35.2)
	35 yrs and over	127 (35.4)
**Place of** **residence**	Urban	143 (37.2)
	Rural	241 (62,8)
**Educational** **level of the** **woman**	Illiterate	113 (29,4)
	Primary	195 (50,8)
	Secondary	76 (19,8)
**Educational** **level of the** **couple**	lliterate couple	136 (35,4)
	One of the spouses literate	141 (36,7)
	Literate couple	107 (27,9)
**Marital status**	Married	369 (96,1)
	Not married	15 (3,9)
**Family income**	< GIMW†	247 (64,3)
	≥ GIMW	137 (35,7)
**Health** **insurance**	Yes	212 (55,2)
	No	172 (44,8)
**Distance from** **the care** **structure**	< ou = 3km‡	76 (19,8)
	>3km and < or = 6 km	195 (50,8)
	>6 km	113 (29,4)
**Age of first** **sexual** **intercourse**	Less than 18 years	364 (94,8)
	More than 18 years	20 (5,2)

† Guaranteed Interprofessional Minimum Wage (=2 828.71 Moroccan Dirham)

‡ Kilometers

Family income is calculated on the basis of the husband's and wife's income, but the proportion of women with a socio-professional activity at the time of the survey is only 11.7%. Consequently, the women studied are divided into two different groups according to the value of the legal minimum wage (SMIG). In 2022, in Morocco, the value of the SMIG was 2,828.71 dirhams (312.95 USD) (18). The first category of relatively low socioeconomic level is composed of families whose monthly income is less than or equal to the SMIG. The second category is composed of families with a medium to high socio-economic level and includes women from families with a monthly income above the SMIG. Therefore, 64.3% of the respondents have a monthly salary below the SMIG. Regarding the current pregnancy, 49.2% had a pregnancy age between 15 and 30 weeks of amenorrhea. Thirty (2.8%) had a history of obstetrical problems, including abortion (5.2%), stillbirth (3.4%) and premature delivery (4.2%).

**Knowledge of the women surveyed on the prevention of MTCT of HIV/AIDS:** In this study, twelve questions were given to pregnant women to measure their knowledge about HIV and the prevention of mother-to-child transmission of HIV. If the score of the answers was lower than the average of the correct answers, which is six correct answers, we speak of low knowledge. Low knowledge of how to prevent MTCT in pregnant women was observed in 75.8% of the women surveyed (Table 2). Low knowledge was significantly associated (p<0.05) with education (86.7% were illiterate), family income (82.2% had an income < SMIG), distance from the nearest healthcare facility (69.7% lived more than>6 km away), source of information (73.9% of those who had high knowledge said their source of information was healthcare professionals), and number of children.

**Table 2:** Distribution of pregnant women by knowledge of prevention of mother-to-child transmission of HIV/AIDS and associated factors

Variables	Modalities	Low knowledge (%)	High knowledge (%)	χ^2^
**Age groups**	15-24 years old	73,5	26,5	1,221§
	25-34 years old	76,3	23,7	
	35 years and over	76,4	23,6	
**Educational level** **of the woman**	Illiterate	86,7	13,3	10,576**
	Primary	71,8	28,2	
	Secondary	69,7	30,3	
**Marital status**	Married	75,5	24,5	0,830§
	Not married	80,8	19,2	
**Family income**	< GIMW†	82,2	17,8	13,760***
	≥ GIMW	65,5	34,5	
**Social security** **coverage**	Yes	78,2	21,8	1,365§
	No	73,0	27,0	
**Distance from the** **care structure**	< ou = 3km‡	86,7	13,3	11,470**
	>3km and < or = 6 km	71,8	28,2	
	>6 km	69,7	30,3	
***Source of*** ***information***	School	86,1	13,9	211,97***
	Health professional	26,1	73,9	
	Medias	97,1	2,9	
	Family/friends	98,0	2,0	
***Obstetrical history***	Abortion	75,0	25,0	1,520§
	Preterm delivery	69,2	30,8	
	Fetal death in utero	87,5	12,5	
	None	75,5	24,5	
***Parity***	Nulliparous	96,2	3,8	109,024***
	1-2 children	88,2	11,8	
	3 children or more	34,9	65,1	

† Guaranteed Interprofessional Minimum Wage (=2 828.71 Moroccan Dirham)

‡ Kilomètres

§ p>0,05

* p<0,05

** p<0,01

*** p<0,001

**Women's attitudes towards the prevention of MTCT for HIV/AIDS**: In this study, ten questions were given to women to measure their attitudes towards HIV infection and the prevention of mother-to-child transmission of HIV. When the score was lower than the average of five correct answers, it was called a “negative attitude” and if it was higher or equal, it was called a “positive attitude”. Marital status (p = 0.027 <0.05), source of information (p = 0.000 <0.05), and parity (p = 0.000 <0.05) were associated with negative attitudes (Table 3).

**Table 3:** Distribution of pregnant women according to their attitudes towards the prevention of mother-to-child transmission of HIV/AIDS and associated factors

Variables	Modalités	Negative attitudes (%)	Positive attitudes (%)	χ^2^
**Age groups**	15-24 years old	69	31	3,927§
	25-34 years old	78,5	21,5	
	35 years and over	69,3	30,7	
**Educational level of** **the woman**	Illiterate	69,0	31,0	1,074§
	Primary	74,4	25,6	
	Secondary	73,7	26,3	
**Marital status**	Married	74,3	25,7	10,998*
	Not married	69,2	30,8	
**Family income**	< GIMW†	72,0	28,0	0,119§
	≥ GIMW	73,6	26,4	
**Social security** **coverage**	Yes	75,7	24,3	2,111§
	No	69,1	30,9	
**Distance from the** **care structure**	< ou = 3km‡	69	31	1,074§
	>3km and < or = 6 km	74,4	25,6	
	>6 km	73,7	26,3	
***Source of*** ***information***	School	83,3	16,7	91,035***
	Health professional	38,7	61,3	
	Medias	85,3	14,7	
	Family/friends	89,1	10,9	
***Obstetrical history***	Abortion	65	35	5,272§
	Preterm delivery	69,2	30,8	
	Fetal death in utero	50	50	
	None	74,3	25,7	
***Parity***	Nulliparous	92,5	7,5	46,464***
	1-2 children	77,7	22,3	
	3 children or more	45,3	54,7	

† Guaranteed Interprofessional Minimum Wage (=2 828.71 Moroccan Dirham)

‡ Kilométres

§ p>0,05

* p<0,05

** p<0,01

*** p<0,001

**Correlation between knowledge and attitudes towards prevention of MTCT of HIV/AIDS**: Table 4 shows that the correlation between knowledge and attitudes was positive (r = 0.529). It also shows that the p-value is below the significance level set in this study (p = 0.000 < 0.05). Thus, there is a significant association between the knowledge and attitudes of pregnant women towards measures to prevent mother-to-child transmission of HIV/AIDS in the province of Essaouira in Morocco.

**Table 4:** Test of correlation between pregnant women's knowledge and attitudes towards prevention of HIV/AIDS MTCT

Variables	N	Mean	Standard deviation	r	p-value
Knowledge	384	1,24	0,429	0,526	0,000**
Attitudes	384	1,27	0,446		

r= Pearson correlation

** Correlation is significant at the 0.05 level (two-tailed)

## Discussion

The results of this study provide important information about knowledge and attitudes regarding HIV/AIDS MTCT prevention measures, particularly among pregnant women in the province of Essaouira, Morocco. In this sense, 75.8% of the pregnant women had low knowledge of HIV/MTCT prevention, with a significant association between level of education (p = 0.005), family income (p = 0.000), and distance from the healthcare facility (p = 0.005).

Moreover, Essaouira is Morocco's fifth-poorest province. Almost forty-nine percent of people are illiterate.

Its economy is mostly dependent on tourism, handicrafts, and fishing (20). In a poll of pregnant women, 70.6% lived more than three kilometers (19.8% more than six kilometers) from the nearest health facility. In actuality, the province's health infrastructure consists of 68 urban and rural primary healthcare facilities, a solitary hospital with 310 functional beds, and a sparsely developed private sector (21).

These low levels of knowledge of HIV/AIDS MTCT corroborate with findings from pregnant women in some areas of Pakistan (22), Nigeria (23), Ethiopia (24), Eswatini (25), Brazzaville (26), Poland (27), and other countries (28) that showed that participants had insufficient knowledge of measures to prevent HIV/AIDS MTCT and poor attitudes towards PMTCT services, respectively. In Morocco, a study conducted in a Moroccan tertiary hospital on nurses' knowledge, attitudes, and practices (KAP) related to HIV/AIDS disease revealed gaps in HIV/AIDS-related KAP (17).

However, knowledge and awareness of HIV transmission and prevention can reduce HIV MTCT by improving PMTCT service-seeking behavior and utilization by women living with HIV when they become pregnant (29).

Nevertheless, other studies have recorded significant knowledge of HIV PMTCT measures among pregnant women in Ethiopia (30), Togo (31), Nigeria (32), Tanzania (33), and Western Ethiopia (34). Negative attitudes towards prevention of HIV MTCT in 72.7% of cases. These results are consistent with those of other studies in Nigeria (23), which reported that 71.21% of pregnant women had negative attitudes towards HIV MTCT prevention, and in Morocco (17). However, other studies have recorded positive attitudes towards prevention of HIV/MTCT in different parts Ethiopia (35), and Nigeria (36). Some research has shown a significant association between knowledge and positive attitudes of pregnant women about PMTCT in Nigeria (36), Iran (37), and Congo (9). In the current study, a significant association was found between knowledge and attitudes (p=0.000) and other socio-demographic factors, such as pregnant women's educational attainment, family income, distance to the nearest medical facility, parity, and the information source (p=0.000). These results are consistent with those from Ethiopia, which indicated that knowledge is strongly influenced by age, education level, employment status, number of antenatal visits for the current pregnancy, and parity (38). PMTCT should be provided to all women of childbearing age to reduce HIV epidemic among newborns (1,9,38). This study corroborates with data from other studies regarding the effectiveness of PMTCT in achieving an HIV-free generation.

Low knowledge scores and negative attitudes were recorded when respondents were interviewed. These low scores reflected a need for information and education by health professionals.

In conclusion, the study showed that a small number of participants had sufficient knowledge and positive attitudes about HIV/MTCT prevention. In contrast, education level, standard of living, number of children, and source of information had statistically significant correlations with pregnant women's knowledge and attitudes. In this sense, it seems necessary to sensitize women of childbearing age to monitor pregnancies and deliveries in order to reduce HIV transmission, especially in public health facilities.

Therefore, it is necessary to provide the different health structures with means of early detection of HIV, to decentralize the supply of antiretroviral drugs, and to provide the means of complete management in the different provinces. Thus, at the level of these structures, it is important to increase awareness of HIV prevention by insisting on the adherence of the entire population to HIV testing in order to reduce morbidity and mortality related to HIV/AIDS in the world and particularly in Morocco. Other studies can be envisaged on the subject in the general population.
